# Advancing Regulatory Science With Computational Modeling for Medical Devices at the FDA's Office of Science and Engineering Laboratories

**DOI:** 10.3389/fmed.2018.00241

**Published:** 2018-09-25

**Authors:** Tina M. Morrison, Pras Pathmanathan, Mariam Adwan, Edward Margerrison

**Affiliations:** Office of Science and Engineering Laboratories, Center for Devices and Radiological Health, U.S. Food and Drug Administration, Silver Spring, MD, United States

**Keywords:** medical devices, computational modeling, regulatory science, virtual patients, virtual clinical trials, FDA

## Abstract

Protecting and promoting public health is the mission of the U.S. Food and Drug Administration (FDA). FDA's Center for Devices and Radiological Health (CDRH), which regulates medical devices marketed in the U.S., envisions itself as the world's leader in medical device innovation and regulatory science–the development of new methods, standards, and approaches to assess the safety, efficacy, quality, and performance of medical devices. Traditionally, bench testing, animal studies, and clinical trials have been the main sources of evidence for getting medical devices on the market in the U.S. In recent years, however, computational modeling has become an increasingly powerful tool for evaluating medical devices, complementing bench, animal and clinical methods. Moreover, computational modeling methods are increasingly being used within software platforms, serving as clinical decision support tools, and are being embedded in medical devices. Because of its reach and huge potential, computational modeling has been identified as a priority by CDRH, and indeed by FDA's leadership. Therefore, the Office of Science and Engineering Laboratories (OSEL)—the research arm of CDRH—has committed significant resources to transforming computational modeling from a valuable scientific tool to a valuable regulatory tool, and developing mechanisms to rely more on digital evidence in place of other evidence. This article introduces the role of computational modeling for medical devices, describes OSEL's ongoing research, and overviews how evidence from computational modeling (i.e., digital evidence) has been used in regulatory submissions by industry to CDRH in recent years. It concludes by discussing the potential future role for computational modeling and digital evidence in medical devices.

## Introduction

The mission of the U. S. Food and Drug Administration (FDA) is to protect and promote public health, and it does so by ensuring the safety, effectiveness and security of FDA-regulated products^1^ These products include, but are not limited to, medical devices, drugs for humans and animals, and biological products such as vaccines and the blood supply, each of which are managed by separate Centers within the Agency. The FDA accomplishes its mission by performing pre-market clearance, approval and post-market monitoring of the performance and safety of products, enforcing, and ensuring compliance to manufacturing processes and quality control, and conducting regulatory science research. The latter, although less well-known in the scientific community, is fundamental to support science-based regulatory decision-making by FDA. Regulatory science encompasses the development of new methods, standards, and approaches to assess the safety, efficacy, quality, and performance of FDA-regulated products and products under development. Each Center in the FDA is committed to advancing these efforts, which have accelerated the product development pathway and regulatory review cycle so that new, innovative products can be made available to the American public.

The FDA faces many challenges (1), such as new and evolving public health threats; rapid scientific breakthroughs and emerging technologies resulting in novel products that may raise unique testing and safety issues; globalization of public health, science, manufacturing and supply chains; and providing timely, accurate and useful consumer information in an age of information overload. To enable the Agency to meet today's public health needs and to be fully prepared for the challenges and opportunities of tomorrow, FDA leadership developed a strategic plan identifying nine target areas, stating that investment in these areas is essential to mission success^2^ Of those nine, four priority areas identified an important role for computational modeling^3^, see Table 1. These priorities also have relevant aspects related to medical devices^4^. regulated by the Center for Devices and Radiological Health (CDRH)^5^, as mentioned by the FDA Commissioner in a blog posted in July 2017^6^.

**Table 1:** In 2011, FDA identified an important role for computational modeling in its strategic priorities.

**The four strategic areas with a specific call for computational modeling**	**Relevance to medical devices**	**Proposed computational modeling methods and approaches**
1. Modernize Toxicology to Enhance Safety	Improving medical device safety; analyzing medical device performance	• (Q)SAR^a^ models to predict the risk to human due to exposure to molecules
2. Stimulate Innovation in Clinical Evaluations and Personalized Medicine to Improve Product Development and Patient Outcomes	Improving health of pediatric and other special populations; identifying new sources of evidence for clinical evaluation	• Computer models of cells, organs, and systems to better predict product safety and efficacy
3. Ensure FDA Readiness to Evaluate Innovative Emerging Technologies	Advancing innovation and evaluating new and emerging technologies	• Virtual physiological patients for testing medical products
4. Harness Diverse Data through Information Sciences to Improve Health Outcomes	Developing novel ways to use clinical data in evaluating medical devices	• Clinical trial simulations that reveal interactions between therapeutic effects, patient characteristics, and disease variables
		• Knowledge building tools: data mining, machine and deep learning, visualization, knowledge bases, high throughput methods
		• Mechanism for sharing and reuse of data, models, and algorithms.

*FDA's Center for Devices and Radiological Health also published a special report on regulatory science^5^ to align with these efforts. The table highlights the priority areas on the left, the medical device relevance in the middle, and the proposed methods and approaches on the right*.

a *Note that (Q)SAR models are classification models that relate the structure of a chemical to its activity, i.e., quantitative structure activity relationship*.

**Figure 1:**
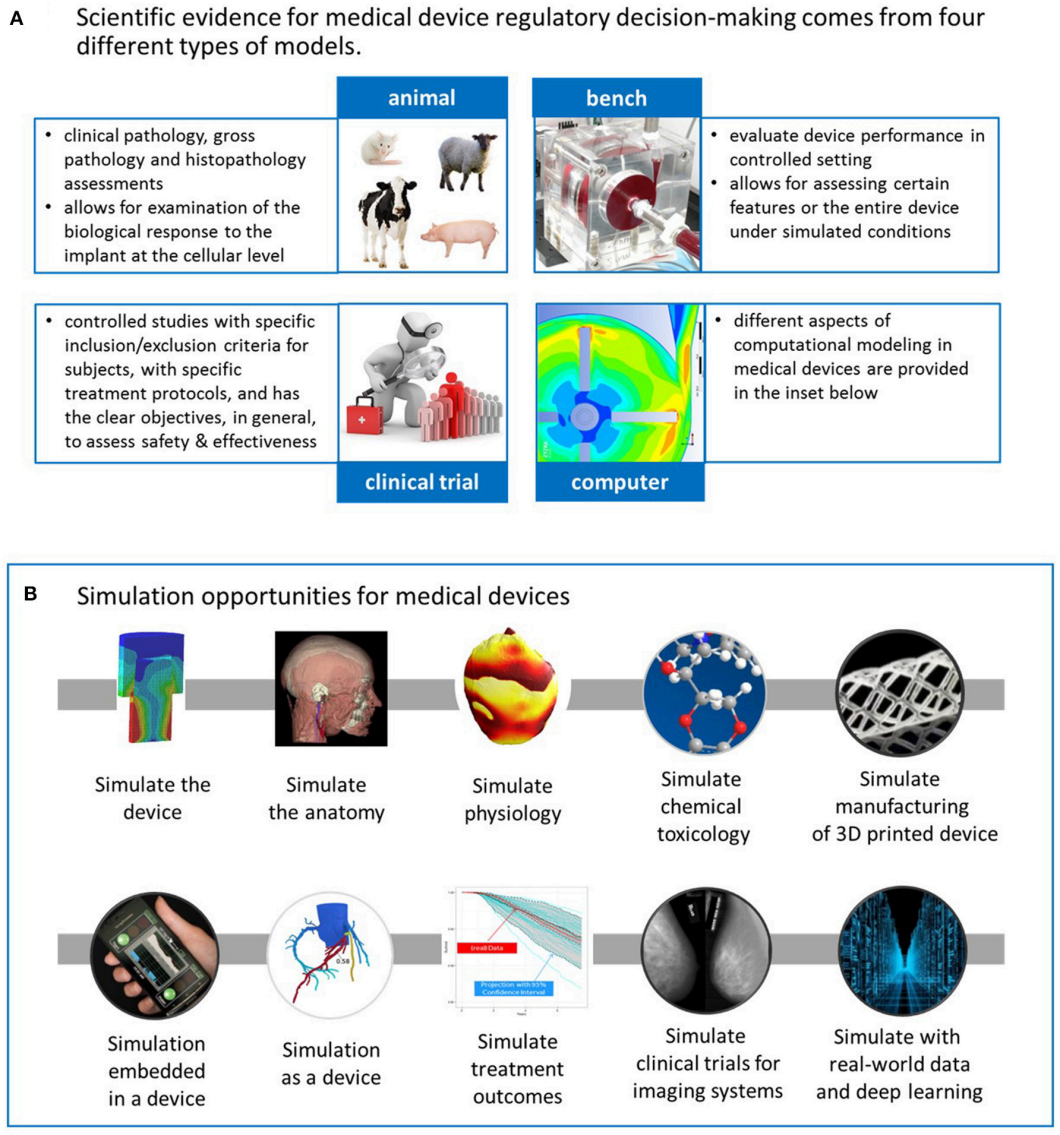
**(A)** CDRH's science-based regulatory decisions about medical devices are made with evidence collected from four different models: animal, bench, computational, and human (i.e., clinical trials). **(B)** Computational modeling has the potential to transform medical device design and evaluation in several ways. The upper row consists of applications that typically support the design or evaluation of the physical device. The lower row represents other applications, such as those embedded in a device or simulation as a medical device. Moreover, computational modeling can also simulate treatment outcomes or simulate the clinical trial for imaging systems. Lastly, it can play a critical role in the development of data-driven models from real-world data. See the text for more details.

CDRH's mission goes beyond protecting and public health; with a vision to be the world's leader in medical device innovation, they provide consumers, patients, their caregivers, and providers with understandable and accessible science-based information about the products it oversees, and facilitate innovation by advancing regulatory science. Science-based regulatory decisions are made with evidence collected from four different models: animal, bench, computational^7^, and human (i.e., clinical trials), see Figure 1A. While each model has its advantages and limitations for evaluating different aspects of medical device performance (3), computational modeling is a promising one for supporting the future of medical devices and healthcare. FDA's Office of Science and Engineering Laboratories (OSEL) has committed significant resources for transforming computational modeling from a valuable scientific tool to a valuable regulatory tool because of its potential for significant cost-savings in evaluating medical devices, simulating performance under scenarios that may not be possible with human use or that could more effectively be evaluated with simulation.

OSEL has a unique role in medical device regulation serving as the research arm for CDRH. OSEL's expertise spans a variety of scientific, engineering, and mathematical disciplines^8^, with a diverse group of 130 full time scientists and engineers (supported by numerous post-doctoral fellows and interns) that provide expert support internally to the regulatory teams and externally to industry, clinical and the scientific communities. They conduct cutting-edge research, ensure readiness for emerging and innovative medical technologies, develop evaluation strategies and testing standards, create accessible and understandable public health information, deliver timely decisions for products across their life cycle, and readily share data and engage with stakeholders to advance regulatory science. The growing area of computational modeling is fully supported by OSEL and CDRH through research and development of methods and tools, serving as expert consultants by reviewing and assessing computational modeling submitted by medical device companies, i.e., sponsors, in regulatory submissions, and publicly sharing computational modeling that supports regulatory decision-making. Moreover, OSEL houses a high-performance computing center which supports scientific computing needs for CDRH and other Centers across FDA^9^

Because OSEL scientists have different roles to support CDRH, we designed and conducted a 35-question survey to better understand computational modeling in research and regulatory domains, including goals and objectives on the use of computational modeling in research, reliance of evidence from computational modeling and simulation (i.e., digital evidence) in regulatory submissions, and opportunities for the future with computational modeling and simulation. Thirty-six OSEL scientists with direct involvement in computational modeling projects and initiatives were interviewed, their responses were transcribed in a database and then shared with the scientists for fact checking. This perspective will present the results from that survey and highlight the different roles that computational modeling has and can play in medical devices, and discuss the potential future for digital evidence and simulation in medical devices.

## Overview of computational modeling for medical devices

Computational modeling can be used to simulate and better understand medical devices in several ways, as depicted in Figure 1B. Starting with the upper row, the simplest and most common implementation of computational modeling for medical devices is simply to simulate the device under a variety of conditions that mimic some aspect of the clinical or use environment to investigate some aspect of the device's performance. Computational modeling applications also include simulating the anatomy or serving as computational human phantoms for medical imaging systems or as a platform for assessing implanted devices; simulating physiology, such as electrophysiology during arrhythmias in the heart, or of pancreatic function; simulating chemical toxicology (using (Q)SAR models), which can support our ability to understand whether or not compounds released from medical devices, such as from dyes and coatings, are harmful; simulating the additive manufacturing process to optimize a 3D-printed product or simulating the substrate on which a 3D-printed product will be manufactured. The applications in the upper row of Figure 1B typically support design or evaluation of a physical, medical device. Other applications, depicted in the lower row, include computational algorithms embedded in a medical device or serving as the medical device, i.e., software as a medical device (4). An example of the former is embedded control algorithms in glucose monitors, which have the potential for advancing modern artificial pancreas systems^10^ used in glucose regulation for patients with diabetes (5). The models for the artificial pancreas have been used to replace *in vivo* animal studies to initiate clinical studies for these closed-loop devices (6). An example of computational modeling as a medical device is the use of personalized simulation to indicate whether a patient is a candidate for a medical device or a pharmaceutical, for example, to simulate an invasive clinical procedure or dosage effect to predict an outcome before the therapy is selected.

Computational modeling can also be used to simulate treatment outcomes. Statistical models have long been used to simulate clinical trial design and interpret results. An evolving concept is that of “virtual patients,” and new statistical models to augment clinical trial design with virtual patients to predict treatment outcomes (7, 8). It is important to note that a “virtual patient” is not necessarily a digitized patient; it is an approach that allows previously collected evidence (such as digital evidence or other historical clinical evidence typically referred to as “external evidence”) to inform the collection of *new* evidence from a clinical trial using Bayesian methodologies. Thus, computational modeling can enable a pathway to expose fewer patients to experimental therapies by relying on other sources of evidence. It can offer an opportunity to address questions that we cannot address clinically due to financial or ethical considerations, and investigate aspects of device performance in many more clinically-relevant cases (hundreds of thousands as compared to hundreds). Computational modeling can facilitate the exploration of using a medical device in populations that cannot be investigated clinically, such as in patients with rare diseases or pediatric patients, without harm. Computational modeling has also enabled the complete “*in silico*” simulation of clinical trials for medical imaging systems. By this we mean the implementation of different computational models to simulate the *entire* clinical evaluation of an imaging system, creating a “virtual clinical trial,” where no patients are physically exposed to the imaging system–more on this later. Lastly, knowledge-base tools can be harnessed to develop data-driven models from big data sources, such as real-world data, and employ deep learning methods to gain relevant insights about medical device use and performance. Computational modeling for medical devices has a broad scope impacting many facets of the product lifecycle, and scientists from OSEL are leading or closely collaborating with leaders in the field in each of the aforementioned categories.

## Computational modeling research

The research conducted in OSEL is directly motivated by regulatory needs, scientific questions arising from the review of regulatory submissions and anticipating future direction of industry needs through technology forecasting. Moreover, the vision of CDRH is to ensure patients in the U.S. have access to innovative medical devices first in the world, and computational modeling is one tool to support faster more efficient regulatory approvals without sacrificing patient safety or the confidence in regulatory decisions. Some companies have stated that the cost for clinical trials may soon outpace revenue (9), and industry will therefore need other relevant and reliable data sources for demonstrating safety and effectiveness of medical devices; computational modeling is a practical and viable method for gathering clinical information to augment clinical trials (10). More details on this are provided in the closing section.

There is a broad range of modeling disciplines that OSEL scientists are using in their medical device-driven research, including photon transport, fluid dynamics, heat transfer, electromagnetism, solid mechanics, acoustics and optics, along with anatomical, physiological, and mechanistic modeling. Other include (Q)SAR models for assessing molecular carcinogenicity (11), deep learning methods and artificial intelligence for analyzing and synthesizing real-world data. Within this diverse range, OSEL has been advancing different areas of computational modeling for medical devices. The following examples provide a glimpse of the many computational modeling applications in OSEL.

Scientists have developed *computational models of medical devices* for investigating a specific approach or consideration about the medical device. It is important to emphasize that the computational studies below are not of a specific manufacturer's device, but of generic devices where the study results have broad impact in that device domain and are translatable to other domains. These models include implantable cardiovascular stents for assessing different methods to calculate fatigue safety factor (12); heart valves implanted with non-circular configurations (13) to assess the impact on stresses and strains; inferior vena cava filters to demonstrate a new method for computing embolus transport (14); hip implants for evaluating the impact of the design on contact mechanics (15); radiofrequency coils for MRI systems (16, 17) to investigate the design parameters on the electromagnetic field; surgical facemasks (18) for evaluating aerosol leakage of different designs; blood pump (19) for assessing the ability to predict hemolysis using computational fluid dynamics; and electrical stimulation of implanted lead wires (20) to investigate local heating. They have also developed new methods for simulating photon transport of x-ray emitters (21) and compressive sensing for imaging systems (22). Another computational effort was the development of a complex constitutive models for absorbable polymers used in medical implants (23).

*Computational models of anatomy or physiology* include improved drug delivery in the cornea with ultrasound energy (24); physiological models of heart cells (25), renal circulation (26), hemodynamic responses to blood volume perturbations (27), left bundle branch block (28), gas dynamics in the retina (29), coupled electrical and mechanical activity in the heart (30); energy absorption in patients with deep-brain stimulators (31–34), breast tissue expanders (35), in pregnant women and fetus during MRI exams (36); subthalamic nucleus (37), the breast (38), cancellous bone (39), the head (40) and whole body models (41, 42).

A part of OSEL's mission is to improve CDRH's ability to evaluate medical devices and support the regulatory approval of innovative medical devices more efficiently without sacrificing safety. Therefore, some of the research efforts involve demonstrating through examples that the output from *computational modeling is a viable source of regulatory-grade evidence*, i.e., sufficiently-credible digital evidence that can support regulatory applications (43–45); developing frameworks (46, 47) and metrics (48) for assessing the trustworthiness of models, and studying workflows for creating reproducible models^11^ (48), and identifying considerations for computational patient models for autonomous medical devices (49).

Other *computational tools to assess specific aspects of device performance* or safety that industry can employ include a simulator for high-intensity focused ultrasound (HIFU) beams and heating effects (50, 51), benchmarks models for computational fluid dynamics (19), patient-specific workflows for assessing clot trapping efficiency in IVC filters (52), surrogate models for predicting device-specific and species-specific hemolysis (Craven et al., under review), optical-thermal light-tissue interactions for photoacoustic breast imaging (53), and an online app for assessing the safety of color additives (54).

Additional efforts are pushing the *state of the art of simulation for medical devices*, including fluid-structure-interaction of deformable blood clots, computational human phantoms for active implants (37), lesion insertion and image reconstruction (55), computational patient models for closed-loop control devices (27), whole-heart modeling for electrophysiology devices (30), computational modeling for determining hemolysis levels in patients supported by blood-circulating medical devices (56), evaluating exposure risk from nickel leaching devices (57) and risk assessment for framing policy and deciding on the stockpile of personal protective equipment for wide-spread outbreaks or virus epidemics (58).

Lastly, as previously mentioned, one team is developing and validating a framework for streamlining the market entry of imaging systems relying solely on *simulation in place of clinical trials*. The VICTRE project (virtual imaging of clinical trials for regulatory evaluation) approach involves simulating the anatomical structure of the breast (with or without a neoplastic lesion), the radiological transmission (i.e., imaging system) and reconstructed images, and the clinical reader studies. By simulating each component of the clinical trial process, there is the potential for minimizing the need for clinical trials and thus the regulatory review of imaging systems (59). Note that the VICTRE project uses statistical analysis tools that evaluate the diagnostic performance of radiologists (virtual or human). An important aspect of the statistical analyses is that they account for radiologist variability and case variability. Such analyses are not trivial and have been developed by OSEL scientists (60–63) with validation based on sophisticated simulation tools (64).

OSEL scientists share their models and data with the public to facilitate the use and broader adoption of these computational tools and approaches. For example, anatomical models of the head (the MIDA model) and whole body models (the Virtual Family) can be downloaded from the IT'IS Foundation's website^12^^,^ ^13^ The experimental and computational data from an FDA-led multi-laboratory study for fluid dynamics on generic medical devices can be found here;^14^ the simulation and statistical tools for the VICTRE project are here^15^ and here (65, 66); the HIFU simulator here^16^ Other software applications are being shared through Github^17^, such as the design of a generic inferior vena cava filter^18^ and a risk assessment tool for assessing color additives^19^

## Computational modeling in regulatory submissions

The OSEL scientists also serve as expert consultants on regulatory submissions. The review and decision about a medical device regulatory application requires a team of experts led by the regulatory offices in CDRH. OSEL scientists serve as specialized, technical experts on the regulatory teams. More than 2500 consulting reviews were completed by all scientists in OSEL in 2017, and about 500 were completed by the 36 scientists interviewed for this perspective. Of the 500 consults performed by the scientists surveyed, 220 (44%) included computational modeling and digital evidence in the submission. (Note that therefore 9% of *all* expert consults performed by OSEL for the regulatory offices in 2017 involved computational modeling). Of these 220, the submission-type breakdown is as follows: 36% were for premarket 510(k) notifications (for moderate risk devices), 25% for clinical trial applications, 24% for pre-submissions, 13% for premarket approval applications (for high risk devices), with only a handful for other submission types. With respect to medical areas, the largest number of consults in 2017 were for neurological devices, followed by cardiovascular and orthopedic devices, imaging systems, and surgical devices.

The survey results indicated that the primary use of computational modeling in regulatory submissions was to identify the appropriate bench testing configurations, such as worst-case or clinically challenging conditions, for cardiovascular, orthopedic, and surgical implants. The second most common use of computational modeling was to provide evidence supporting the safety assessment of patients with and without implanted devices when exposed to the radiofrequency (RF) fields of an MR system. A noteworthy example of the latter is the recent clearance^20^ of the first 7 Tesla MRI system (Siemens Magnetom)^21^, where the Virtual Family (41) and the MIDA head model (40) were used to predict aspects of safety and effectiveness of the new system. Examples of the former include RF safety evaluation for patients with implanted electrically passive (e.g., joint replacement, stents) or electrically active devices (e.g., neurostimulators, pacemakers, cochlear implants). Other modeling examples include therapeutic ultrasound systems where simulation results of the ultrasound energy delivered to *in vivo* locations have been used in regulatory submissions as justification for system parameters, or the recent clearance of Compressed Sensing GRASP-VIBE® to support high-resolution dynamic abdominal imaging under free-breathing. From the 510(k) summary^22^, “A comparison of the functionality was performed between the new feature and the device feature by detailed simulations with a numerical [computational] phantom”.

In general, computational modeling can be part of a regulatory submission in two ways. The first is when simulation results serve as supporting (digital) evidence in a marketing application for a medical device. The second is when simulation is a medical device, such as for clinical decision support; this is “software as a medical device.” Virtually all consults regarding computational modeling were of the former; the latter, with just a handful of submissions, is a new growth area for CDRH, especially with the release of the FDA guidance that describes the clinical evaluation for these software application, and the new program area on Digital Health Technologies^23^ Two examples of software as a medical device that have received FDA clearance used patient-specific computational models generated from CT imaging data to non-invasively predict clinically-relevant quantities for treatment selection. Heartflow® generates a personalized 3D model of the patient's coronary arteries and simulates blood flow to predict fractional flow reserve^24^ The CardioInsight® Mapping System generates a personalized model of the patient's heart and torso, then simulates the electrical activity on the heart surface from body surface potential recordings^25^ For more information on these and other patient-specific cardiovascular models see (67).

As indicated by the relatively large number of pre-submissions (approximately five dozen) that contain computational modeling, many sponsors are using this mechanism to discuss with FDA how their computational approaches will be used in different regulatory pathways. The pre-submission^26^ process enables interaction between companies and FDA to discuss issues (e.g., outstanding regulatory deficiencies) or present new technology or regulatory approaches. Pre-submissions might include details describing how computational modeling might support device performance, augment clinical trials, or be a part of a software as a medical device, but might also include the introduction of innovative devices for informational purposes or for strategic regulatory planning. The mechanism for early interaction is called the *Information Meeting*, found on page 22 of the guidance.

In this section, we have presented some success stories of computational modeling being used to support medical device regulatory review. However, there remain hurdles for broader adoption of computational modeling. FDA is using its leadership role to help overcome some of these hurdles; one in particular is on communication. The regulatory review process is typically dominated by the review of tens (sometime hundreds) of test reports. CDRH reviewers do not conduct or run simulations for specific regulatory submissions, and consequently rely on the details of a report to understand what was accomplished. Detailed and comprehensive reporting of computational modeling can thus substantively improve the acceptance of digital evidence submitted to CDRH. In 2016, FDA published a guidance document (68) on the details that should be provided to CDRH if computational modeling is used in a regulatory application. The scope of this guidance document, however, does not address the *adequacy* of the evidence, and therefore adherence to the guidance may not always result in a sufficiently credible digital evidence to support the device safety and/or effectiveness claims. Reasons for this failure include the lack of appropriate scope of use for the computational model or that the verification and validation (V&V) results provided do not support using the model for the specified use. Industry has communicated to FDA that what remains unclear is the V&V evidentiary bar and lack of standards for computational modeling studies. Therefore, FDA has been working closely with the ASME V&V40 Standards Subcommittee on a new standard (44) that will be published in Summer 2018. It presents a risk-informed credibility assessment framework that will help decision-makers determine the V&V evidence needed to support using a computational model for a specific context of use. Other device-specific modeling standards are being developed through ASTM and IEEE. Moreover, FDA actively engages with stakeholders about computational modeling efforts by hosting yearly workshops and conferences with co-sponsors such as the NIH & NSF^27^ and the Biomedical Engineering Society (69), recorded webinars^28^^,^ ^29^^,^ ^30^ and training seminars (70) on these documents. The use of simulation and digital evidence is rapidly evolving so FDA hopes industry will connect early and often through the pre-submission process to discuss potential opportunities for their computational modeling approaches.

## Future of computational modeling in medical devices

The rapid advance of technology has drastically changed the power and availability of computational modeling tools. Increased storage capacity via the cloud, the acceleration of the graphics processing unit (GPU), parallelization, multicore machines, and high performance computing have transformed and facilitated the building of higher fidelity models and models with more complexity through multiscale and multiphysics applications, and improved the resolution and capability for enhanced visualization. With this increased capability and power, evidence from computational modeling has the potential to replace traditional, more burdensome data collection from other models. Notable are the simulations of radiofrequency energy absorption that have replaced confirmatory clinical trials for the MR safety assessment of implants previously discussed (10). Wanting to find more opportunities to minimize the burden of animal and human studies, CDRH will continue to promote the use of computational modeling in medical device development, applications and regulatory submissions and is committed to ensuring the appropriate research, methodologies and expertise is available to make such advances. For example, the FDA has been exploring the use of computational modeling as a possible way to facilitate better reprocessing for reusable medical devices^31^ Computational fluid dynamics models have the capability to isolate high risk regions in new device designs and can provide appropriate cleaning protocols to eliminate the risk of infection in reusable devices (71).

Another demonstrative example of FDA's commitment to facilitating innovation in medical devices is the collaboration between CDRH and industry through the Medical Device Innovation Consortium (MDIC) using the mechanism of a “mock submission” to demonstrate the regulatory process and evidence collection that could be submitted to FDA when using a new framework to augment clinical trials with virtual patients^32^. The mock submission approach has been a successful means to gather input from industry and FDA about new and innovative approaches for medical device evaluation (72). The MDIC, a non-profit in the U.S., was established to advance medical device regulatory science for patient benefit. The “Clinical Trials Informed by Bench and Simulation” working group included members from industry and FDA; their main objective was to establish and implement a framework, called the virtual patient discount model (VPDM), for incorporating virtual patients from simulation with real patient data from a clinical study through statistical simulations. Due to its novelty, the members of the working group formed a “sponsor team,” with members from industry and FDA, and prepared the mock submission proposing to initiate a clinical trial with a reduced number of actual patients by harnessing virtual patients; the goal was to present and evaluate the VPDM. The VPDM (73) harnesses Bayesian methods in conjunction with FDA guidance (74) on the use of Bayesian statistics in medical device clinical trials. The mock submission highlighted two key themes. First, early communication between the sponsor and regulatory teams is important to identify areas for detailed discussion, education, or that raise issues of concern, particularly due to the intersection of clinical statistics and engineering simulation. Second, model credibility, suitability, and context of use should be integral to the model development work, as they will heavily influence the success of the approach. All mock submission documents and FDA's formal response are publicly available on the MDIC website for the Virtual Patient Project.

The aforementioned VICTRE project for imaging systems also exemplifies other advances FDA is considering for augmenting clinical trials. The power of this approach lies in the ability to replace expensive and lengthy clinical trials for new imaging systems with completely *in silico* trials. The physics for these systems are well-known and all aspects of the evaluation cycle can be simulated with sufficient confidence to replace each step with simulation. The main objective of this approach is to achieve the *same* regulatory conclusion with the virtual clinical trial as with a large 600-patient clinical trial (75). The success of this approach relies on statistical models, deep learning and artificial intelligence (AI); these advanced analysis and statistical methods, which are comparable with mechanistic, predictive engineering tools, have the potential to dramatically transform medical devices (76). In fact, CDRH just permitted the marketing of the first medical device with AI^33^.

The device uses deep learning algorithms (77) to analyze images of the eye taken with a retinal camera for detecting diabetic retinopathy.

In other industries, computational modeling has long been recognized as a crucial scientific tool for getting innovative products into the hands of consumers^34^ (78) and facilitating design excellence through product lifecycle management (PLM). “PLM is a business solution which aims to streamline the flow of information about the product and related processes throughout the product's lifecycle such that the right information in the right context at the right time can be made available (79)”. If the medical device industry were to embrace PLM, they could more fully harness the power of simulation in each phase of the product's lifecycle and utilize AI tools to implement knowledge gained from real-world data to enhance their understanding of performance, support continuous improvement, and inform new designs and therapies. FDA also believes that computational modeling is poised to become a critical tool for accelerating regulatory decision-making. Continued adoption will be essential for advancing FDA's mission, improving our ability to evaluate medical devices more efficiently, reducing regulatory burden for sponsors, and accelerating the introduction of innovative technologies to the U.S. market for the benefit of patients.

## Author contributions

TM led this study, designed the survey with PP and MA, analyzed results, organized and gathered references, drafted perspective article, organized multiple reviews and connected with authors cited in the article ensure accuracy. MA conducted the survey and interviews, collated responses in a database, and analyzed results. PP co-designed the survey, analyzed results, provided input and edited article. EM provided input on survey, analyzed results, provided input and edited article.

### Conflict of interest statement

The authors declare that the research was conducted in the absence of any commercial or financial relationships that could be construed as a potential conflict of interest. The reviewer AH declared a past co-authorship with one of the authors TM to the handling Editor. The reviewer AH and handling Editor declared their shared affiliation.

## Footnotes

^1^https://www.fda.gov

^2^http://www.fda.gov/ScienceResearch/SpecialTopics/RegulatoryScience/

^3^Computational modeling is the process of representing a real-world system by means of a computer and then running the simulation by implementing a numerical scheme.

^4^The FDA definition of medical devices excludes drugs, which achieve their effects through chemical action within or on the body. The vast majority of medical device manufacturers are classified by the U.S. Department of Commerce in five industries: x-ray and electromedical equipment, surgical and medical instruments, surgical appliances and supplies, dental equipment, and ophthalmic goods (2)

^5^https://www.fda.gov/AboutFDA/CentersOffices/OfficeofMedicalProductsandTobacco/CDRH/CDRHReports/ucm274152.htm

^6^https://blogs.fda.gov/FDAvoice/index.php, post July 7, 2017.

^7^Evidence from algorithms, and computer-based modeling and simulation will be referred to as digital evidence.

^8^https://www.fda.gov/AboutFDA/CentersOffices/OfficeofMedicalProductsandTobacco/CDRH/CDRHOffices/ucm115989.htm

^9^https://scl-wiki-01.fda.gov/wiki/index.php/Main_Page

^10^https://www.fda.gov/MedicalDevices/ProductsandMedicalProcedures/HomeHealthandConsumer/ConsumerProducts/ArtificialPancreas/default.htm

^11^NIBIB. R01EB024573, https://simtk.org/projects/kneehub

^12^The MIDA Model: https://www.itis.ethz.ch/virtual-population/regional-human-models/mida-model/,

^13^The Virtual Family: https://www.itis.ethz.ch/virtual-population/virtual-population/overview/

^14^https://nciphub.org/wiki/FDA_CFD

^15^https://github.com/didsr/victre

^16^https://www.mathworks.com/matlabcentral/fileexchange/30886-high-intensity-focused-ultrasound-simulator.

^17^https://github.com/didsr

^18^https://github.com/kenaycock/Generic-IVC-Filter

^19^https://dsaylor.github.io/CHRIS/

^20^510(k) Summary: https://www.accessdata.fda.gov/cdrh_docs/pdf17/K170840.pdf

^21^https://usa.healthcare.siemens.com/news/magnetomterrafdaclearance.html

^22^510(k) Summary: https://www.accessdata.fda.gov/cdrh_docs/pdf17/K173617.pdf

^23^https://www.fda.gov/medicaldevices/digitalhealth/

^24^https://www.accessdata.fda.gov/cdrh_docs/pdf15/K152733.pdf

^25^https://www.accessdata.fda.gov/cdrh_docs/pdf16/k162440.pdf

^26^FDA Final Guidance, Software as a Medical Device (SAMD): Clinical Evaluation. (2017). Available from: https://www.fda.gov/downloads/medicaldevices/deviceregulationandguidance/guidancedocuments/ucm311176.pdf

^27^https://www.fda.gov/ucm/groups/fdagov-public/@fdagov-meddev-gen/documents/document/ucm364603.pdf

^28^ORS Webinar: FDA - Modeling and Simulation Initiatives at CDRH on Vimeo (2017): https://vimeo.com/194712010,

^29^ORS Webinar: FDA - Assessing the Predictive Capability of Computational Modeling for Medical Device Submissions on Vimeo (2017): https://vimeo.com/221479130,

^30^Xtalks Webinar (2018) FDA Perspectives on Computer Simulations in the Evaluation of Medical Devices, https://xtalks.com/webinars/fda-perspectives-on-computer-simulations-in-the-evaluation-of-medical-devices/

^31^https://www.fda.gov/MedicalDevices/DeviceRegulationandGuidance/ReprocessingofReusableMedicalDevices/ucm252952.htm

^32^Virtual Patient Website, http://mdic.org/computer-modeling/virtual-patients/

^33^https://www.fda.gov/newsevents/newsroom/pressannouncements/ucm604357.htm

^34^UL Science news: https://newscience.ul.com/articles/computational-modeling-of-lithium-ion-batteries
